# An ultrasensitive sorting mechanism for EGF Receptor Endocytosis

**DOI:** 10.1186/1752-0509-2-32

**Published:** 2008-04-07

**Authors:** Hannah Schmidt-Glenewinkel, Ivayla Vacheva, Daniela Hoeller, Ivan Dikic, Roland Eils

**Affiliations:** 1 Division Theoretical Bioinformatics, German Cancer Research Center (DKFZ), 69120 Heidelberg, Germany; 2 Innsbruck Medical University, Biocenter, Medical Biochemistry, A-6020 Innsbruck, Austria; 3 Institute for Biochemistry II, Goethe University Medical School, 60590 Frankfurt, Germany; 4 Institute for Pharmacy and Molecular Biotechnology (IPMB), 69120 Heidelberg, Germany

## Abstract

**Background:**

The Epidermal Growth Factor (EGF) receptor has been shown to internalize via clathrin-independent endocytosis (CIE) in a ligand concentration dependent manner. From a modeling point of view, this resembles an ultrasensitive response, which is the ability of signaling networks to suppress a response for low input values and to increase to a pre-defined level for inputs exceeding a certain threshold. Several mechanisms to generate this behaviour have been described theoretically, the underlying assumptions of which, however, have not been experimentally demonstrated for the EGF receptor internalization network.

**Results:**

Here, we present a mathematical model of receptor sorting into alternative pathways that explains the EGF-concentration dependent response of CIE. The described mechanism involves a saturation effect of the dominant clathrin-dependent endocytosis pathway and implies distinct steady-states into which the system is forced for low vs high EGF stimulations. The model is minimal since no experimentally unjustified reactions or parameter assumptions are imposed. We demonstrate the robustness of the sorting effect for large parameter variations and give an analytic derivation for alternative steady-states that are reached. Further, we describe extensibility of the model to more than two pathways which might play a role in contexts other than receptor internalization.

**Conclusion:**

Our main result is that a scenario where different endocytosis routes consume the same form of receptor corroborates the observation of a clear-cut, stimulus dependent sorting. This is especially important since a receptor modification discriminating between the pathways has not been found experimentally. The model is not restricted to EGF receptor internalization and might account for ultrasensitivity in other cellular contexts.

## Background

Endocytosis is the process by which activated transmembrane receptors are directed into the endosomal system from the plasma membrane [1-4]. In the past years, it has emerged as a powerful mechanism for the cell to temporally and spatially control its signaling response [5]. Ligand induced phosphorylation of EGF receptor creates docking sites for adaptor proteins, such as EPS15, epsin and AP-2 [6,7]. Via direct or indirect binding, adaptors recruit the receptor to special membrane regions which are characterized by a particular composition of cage-proteins and/or -lipids [8,9]. The forming vesicles pinch off the membrane and carry their cargo to distinct intracellular locations, which might account for the specificity of the invoked signal [1,10]. Endocytosis may direct the receptors for lysosomal degradation or recycle them back to the membrane [10-12]. Proper sorting of the EGF receptor into the correct endocytosis route is crucial for cell functioning as indicated by the fact that corruption of the sorting e.g. by viral proteins [13,14] may result in impaired receptor downregulation and increased mitogenic activity [15].

Clathrin-dependent endocytosis (CDE) was the first receptor internalization mechanism to be discovered and is generally considered the major route for EGF receptor (reviewed in [1,5,6,9]). Nevertheless, receptor internalization mechanisms that do not employ the structural protein clathrin, but arise from lipid rafts and caveolin-rich membrane regions exist (Clathrin-independent endocytosis, CIE) [8,9,16,17]. The important question which molecular events govern the sorting of the EGF receptor into the different endocytosis pathways remains unanswered [5,8,9,18-20].

**Figure 1:**
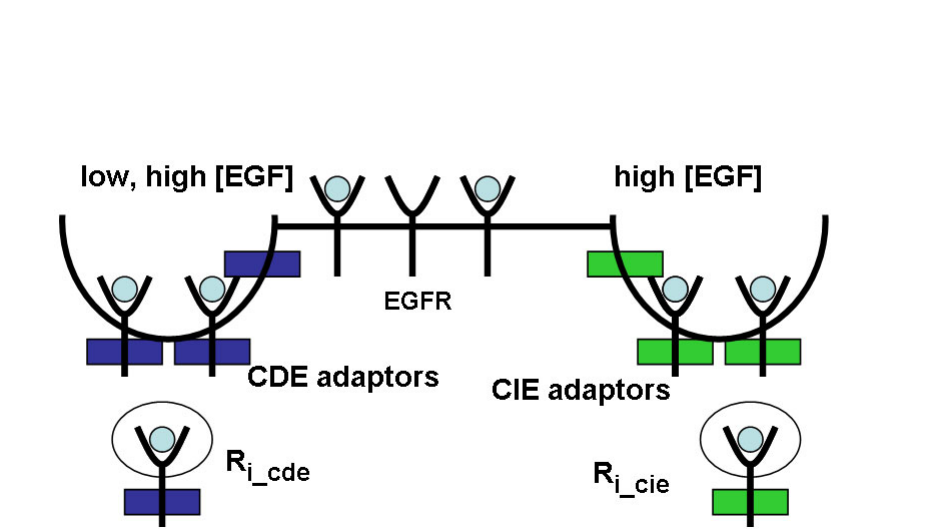
**CDE and CIE pathways of EGF receptor**. An illustration of CDE and CIE pathways of EGF receptor. High EGF concentrations induce CIE, whereas CDE is observed at low and high EGF concentrations. The adaptors for the respective endocytosis pathways are referred to as CDE- or CIE-adaptors, respectively. See list of abbreviations.

A study addressing the sorting between Clathrin- vs lipid raft/Caveolae-mediated Endocytosis in mammalian cells suggested an interesting mechanism for the sorting process [21]: the distribution of receptors into the two pathways was shown to be EGF-concentration dependent. In the presence of low concentrations of EGF, the receptor was exclusively internalized via CDE, whereas at high concentrations, receptors were equally distributed between CDE and CIE (Figure 1).

From a modeling point of view, the behaviour of the clathrin-independent pathway resembles an ultrasensitive response: activation of the pathway is suppressed for low input EGF values, to reach the same level as the clathrin-dependent pathway for high input levels. Theoretically, several different mechanisms can explain ultrasensitive behaviour. Multisite modifications lead to a sigmoidal response of the modified molecule [22-24], an effect that can be enhanced by consecutive arrangement in the form of cascades [25-29] which has also been validated experimentally [30].

Other models of ultrasensitivity have been derived for Michaelis-Menten type enzyme reactions: the presence of a stoichiometric inhibitor of an enzyme can suppress a reaction up to a certain threshold [28]. In (de-)modification cycles ultrasensitivity occurs when the opposing enzymes work in the zero-order regime [31], a mechanism which has been shown to work during morphogen directed pattern formation [32], or if the abundance levels of unmodified substrate and enzyme are sufficiently high [33]. Mathematical modeling has previously played a significant role in elucidating the mechanisms of EGF receptor signaling and endocytosis [34-42]. In a series of quantitative studies the interaction between receptors and endocytosis machinery was evaluated [34,35,38,43]. Here, the existence of at least two distinct internalization pathways with different affinities for the EGF receptor was discovered [35,43]. In [21] it was reported that mono-ubiquitination (mono-Ub) of the EGF Receptor could only be observed at high EGF concentrations, raising the question whether mono-Ub might serve as a discriminative feature, which, when appended to the receptor, selectively targets the receptor to CIE [19,44]. This, however, conflicts with reports on the involvement of ubiquitin-binding adaptor proteins such as epsin and EPS15 during CDE [19,20,45-49].

To address this controversy, we built a mathematical model of the sorting process. We address the functional consequences of different affinities with which internalization pathways are entered and explain how a switch-like response of CIE may result simply from a saturation effect of the CDE pathway. Together with the observation of EGF-concentration dependence of CIE, this analysis invites attention to an ultrasensitive regulatory mechanism for endocytic sorting. We give an analytical derivation of the switch-effect and derive regimes of reaction parameters and initial values for which the switch is preserved. Further, we describe its extensibility to more than two pathways. Importantly, the mechanism imposes only weak assumptions on the underlying interaction structure and parameter values. In summary, we give evidence for the hypothesis that the main purpose of post-ligand binding modifications of the EGF receptor such as ubiquitination does not lie in the discrimination between alternative endocytosis pathways.

## Results

We built a system of ordinary differential equations (ODEs) that models the sorting of EGF receptor into clathrin-dependent or -independent endocytosis pathways. The equations read:

(1.1) d(EGF)/dt = -k_f _* EGF * R + k_r _* R_EGF

(1.2) d(R)/dt = -k_f _* EGF * R + k_r _* R_EGF

(1.3) $$\begin{array}{lll} \text{d}(\text{R\_EGF})/\text{dt} & = & {\text{k}}_{\text{f}}*\text{EGF}*\text{R}-{\text{k}}_{\text{r}}*\text{R\_EGF}\\ & & -{\text{k}}_{\text{cde}}*\text{R\_EGF}*\text{CDE}-{\text{k}}_{\text{cie}}*\text{R\_EGF}*\text{CIE} \end{array}$$

(1.4) d(CDE)/dt) = -k_cde _* R_EGF * CDE

(1.5) d(CIE)/dt) = -k_cie _* R_EGF * CIE

(1.6) d(R_cde_) = -k_cde _* R_EGF * CDE

(1.7) d(R_cie_) = -k_cie _* R_EGF * CIE

The model contains the binding reaction of EGF to receptor R, which leads to one form of activated receptor (R_EGF), capable of entering clathrin-dependent or -independent endocytosis.

In order to simulate the entry of ligand-bound receptor into an endocytosis pathway, we introduced variables CDE and CIE, representing adaptors for clathrin-dependent and -independent endocytosis which R_EGF can enter with rates k_cde _and k_cie_, respectively. The variables CDE and CIE represent the amount of the limiting factor in each pathway, which could be adaptor- or cage-proteins. We assume that the affinity of activated receptors R_EGF is significantly higher for the CDE-pathway (k_cde _≫ k_cie_). To quantify the fraction of receptor going either pathway, we introduced variables R_i_cde _and R_i_cie_. The steady-state values of these variables represent the amount of R_EGF internalized via CDE and CIE, respectively. The model equations were derived according to the law of mass-action [50].

### CIE-internalization depends on number of receptors and strength of EGF-stimulation

**Figure 2:**
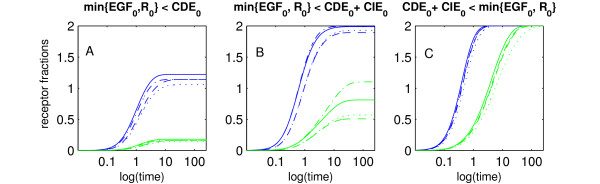
**Temporal Evolution of internalized receptors**. Time trajectories of R_i_cde _(blue) and R_i_cie _(green) for three different classes of initial conditions. In case A (B) at least one of EGF_0 _or R_0 _stays below CDE_0_ (CDE_0 _+ CIE_0_). In case C, both EGF_0 _and R_0 _exceed CDE_0 _+ CIE_0_. Initial values were chosen arbitrarily such that these conditions are satisfied. In all three cases CDE_0 _= 2.0, CIE_0 _= 2.0 and (A) (EGF_0_, R_0_) = (1.2, 1.5), (1.6, 1.3), (2.3, 1.3), (1.4, 2.5); (B) (2.5, 3.0), (3.1, 2.4), (3.1, 4.5), (5.0, 2.8); (C) (4.3, 4.7), (5.0, 5.2), (5.5, 5.0), (5.3, 6.0).

We systematically scanned the space of initial values of the model (equations 1.1 – 1.7) to investigate the effect of EGF stimulation on receptor distribution into CDE or CIE (see Methods). Figure 2 shows the time trajectories of R_i_cde _and R_i_cie _for three different classes of initial conditions, each represented by four different sets of values (see Figure caption). The three classes of initial values have distinct effects on the internalization-behavior of R_EGF, the activated receptor. They represent assumptions on the relative quantities of EGF-molecules, unbound receptors and endocytosis adaptors.

The first class of initial values (Figure 2A) represents the case that either the initial number of unbound receptors (R_0_) or EGF-molecules (EGF_0_) is lower than the capacity of the CDE-pathway (CDE_0_) (Generally, X_0 _denotes the initial value of variable X). This corresponds to an experimental setting where cells are stimulated with low EGF-concentrations, i.e. EGF_0 _< CDE_0_. Initial values from the second class are such that either R_0 _or EGF_0 _are below the capacity of both internalization pathways (CDE_0 _+ CIE_0_) (Figure 2B), whereas the third class reflects the case that both R_0 _and EGF_0 _exceed the capacity of both internalization pathways (Figure 2C).

It can be seen that in case A, R_i_cie_-production stays close to zero. In case B, internalization via CIE does occur, albeit to a lesser degree than CDE. For case C, receptors are equally partitioned between CDE and CIE.

### Conditions on receptor number for switch-effect of CIE-internalization

The simulations shown in Figure 2 suggest conditions on the receptor number under which an EGF-dependent switch of CIE-internalization will occur. If a cell possesses less receptors than CDE-adaptors, CIE-internalization will be low independent of EGF-stimulation (cf. Figure 2A). If the cell exhibits more receptors than adaptors for CDE, but less than for both pathways, then, for EGF-stimulations exceeding CDE_0_, a moderate fraction of receptor will internalize via CIE (cf. Figure 2B). Finally, if the amount of receptors is higher than the combined capacity of both pathways, CIE-internalization will be switched on equally strong as CDE-internalization for EGF-stimulations that are higher than this combined capacity (cf. Figure 2C).

**Figure 3:**
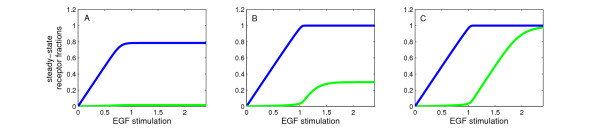
**Steady-State behaviour of internalized receptors**. Plotted are steady-state values of R_i_cde _(blue) and R_i_cie _(green) as a function of EGF-stimulation (EGF_0_). An ultrasensitive response of CIE-internalization with respect to EGF occurs if R_0 _> CDE_0 _(B) or R_0_ > CDE_0_+CIE_0 _(C), but not if R_0 _< CDE_0 _(A). In (B) and (C), when EGF_0_ exceeds the amount of CDE_0_, CIE-internalization switches on abruptly, with a maximal response if (CDE_0_+CIE_0_) < min{CIE_0_, R_0_} (C). In all three cases CDE_0 _= 1.0, CIE_0 _= 1.0 and (A) R_0 _= 0.8, (B) R_0 _= 1.3 and (C) R_0 _= 2.4.

To test this hypothesis, we performed the following virtual experiment. We chose three sets of initial values for receptor R, CDE- and CIE-adaptors such that they fall within the three respective classes: R_0 _< CDE_0_, R_0 _< CDE_0 _+ CIE_0 _or CDE_0 _+ CIE_0 _< R_0_, and stimulated the system with increasing amounts of EGF. Figure 3 shows the steady-state amounts of R_i_cde _(blue) and R_i_cie _(green) as a function of EGF_0_.

As predicted, for receptor levels lower than CDE_0 _(Figure 3A), CIE-internalization stays close to zero, independently of EGF-stimulation. If the initial amount of receptors is greater than the capacity of CDE, CIE-internalization sets in abruptly, albeit to a moderate degree compared to CDE-internalization, for EGF-stimulations greater than CDE_0 _(Figure 3B). Finally, if the initial number of receptors is greater than the capacity of both pathways, the CIE pathway switches on to an equal extent as CDE-internalization (Figure 3C).

We have thus derived an ultrasensitive response of CIE-internalization with respect to EGF-stimulation, without assuming any discriminative receptor modifications. Rather, it is necessary and sufficient that the initial amount of receptors is higher than the capacity of the CDE-pathway (CDE_0 _< R_0_, moderate switch) or both pathways (CDE_0 _+ CIE_0 _< R_0_, maximal switch).

### Correspondence to distinct classes of steady-state

For dynamical systems with multiple steady-states, a certain steady-state will be reached depending on whether the system starts in the corresponding basin of attraction [23,51]. Thus, a switch between steady-states occurs for different vectors of initial values, provided that the separatrix, i.e. the hypersurface between neighboring basins of attraction, is crossed.

We investigated, whether the switch-effect of CIE-internalization corresponds to such a transition between distinct steady-states of the system. Analytically, one derives two classes of steady-states (see Methods for derivation of conditions):

(I) (EGF* = 0 ∧ R* = 0) ∨ R_EGF* = 0,

(II) $${\text{CDE}}^{*}=0\wedge {\text{CIE}}^{*}\text{=0}\wedge {\text{R\_EGF}}^{*}=\frac{{\text{k}}_{\text{f}}}{{\text{k}}_{\text{r}}}{\text{EGF}}^{*}*{\text{R}}^{*},$$

where X* denotes the steady-state concentration of the respective component.

Note that *classes *of steady-states are used since not all variables are assigned specific values. For example, in both cases ${\text{R}}_{\text{i\_cie}}^{*}$ and ${\text{R}}_{\text{i\_cde}}^{*}$ are not uniquely determined and depend on the corresponding initial values. Steady-state class I reflects the case that either all available EGF (EGF* = 0) or all free receptors R (R* = 0) have been absorbed in the binding reaction and all activated receptors R_EGF have been internalized. In steady-state class II neither receptors nor ligand are limiting for the internalization process and have come to an equilibrium with R_EGF. Instead, the capacity of both internalization pathways has been depleted (CIE* = CDE* = 0).

The systematic scan of initial values and subsequent solving of the system until steady-state revealed initial conditions under which each steady-state class is reached. We found that if both EGF-stimulation and initial receptor level are higher than the capacity of both internalization pathways (CDE_0 _+ CIE_0 _< min{R_0_, EGF_0_}, initial value class C), the system tends towards steady-state class II. Otherwise, steady-state class I will be reached. In this case, if EGF-stimulation is below the amount of receptors, all EGF will be depleted in the binding reaction (EGF* = 0), whereas if it is above, all receptors will be consumed (R* = 0).

**Figure 4:**
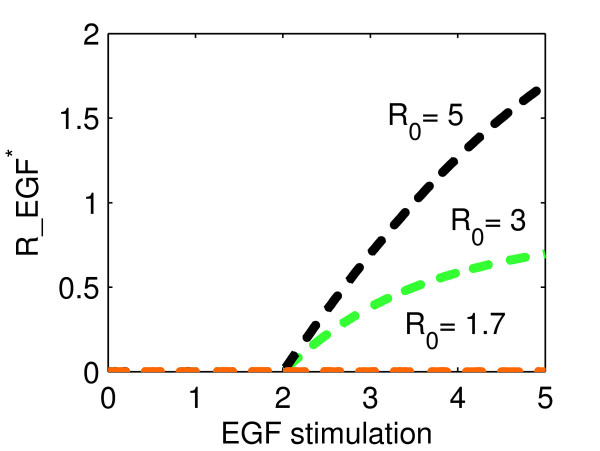
**Dependence on initial values for steady-state**. Plotted are steady-state values of ligand-bound receptor (R_EGF*) as a function of EGF-stimulation for different initial receptor levels R_0_. For R_0 _= 1.7 (orange), i.e. R_0 _< CDE_0 _+ CIE_0_, the system reaches steady-state class I independently of EGF-stimulation, as seen from R_EGF* = 0. If R_0 _= 3 (green) or R_0 _= 5 (black), i.e. CDE_0 _+ CIE_0 _< R_0_, the steady-state value of R_EGF becomes positive for EGF-stimulations higher than 2 (steady-state class II).

This is exemplified in Fig. 4, where the steady-state value of ligand-bound receptor (R_EGF*) is plotted as a function of EGF-stimulation for different initial receptor levels R_0_. Here, CIE_0 _= CDE_0 _= 1. For R_0 _= 1.7 (orange), i.e. R_0 _< CDE_0 _+ CIE_0_, the system reaches steady-state class I independently of EGF-stimulation, as seen from R_EGF* = 0. If R_0 _= 3 (green) or R_0 _= 5(black), i.e. CDE_0 _+ CIE_0 _< R_0_, the steady-state value of R_EGF becomes positive for EGF-stimulations higher than 2 (steady-state class II).

Applying these derived conditions on the initial values, we can also show that the steady-states are stable. A steady-state is stable, if, for small perturbations, the system returns to this steady-state. Consider steady-state class I with R* = 0 and R_EGF* = 0. CIE* and CDE* are not clearly defined in this case, but according to the conditions on the initial values we derived, we know that R_0 _< CDE_0 _+ CIE_0_. This means that in steady-state, at least one of the two adaptor variables must be greater than zero, i.e. 0 = R* < CDE* + CIE*. If we apply a sufficiently small perturbation to the system, and set the obtained value as the new start vector, this last inequality will still hold due to the continuity of the functions. According to the conditions on initial values we derived in the previous paragraph, the system will tend back to R* = 0 and R_EGF* = 0. Hence we showed stability of steady-state class I. An analogous argument can be used to show stability of steady-state class II.

In [21] it was reported that for high ligand concentrations the activated receptor is equally partitioned between CDE and CIE. Assuming similar initial abundance levels of adaptors, our simulations show that this is the case for initial receptor levels higher than the sum of both initial adaptor values (Fig. 2C, Fig. 3C). We thus hypothesize that in cells, where the steady-state levels of internalized receptors via CIE and CDE are similar, the amount of receptors exceeds the capacity of both pathways. In this case, treatment of the cells with low vs high EGF-stimulations, corresponds to a transition of the system between steady-state classes I and II (see Table 1).

**Table 1:** Conditions on initial values for steady-states. Assuming that the initial number of receptors is higher than the combined capacity of both pathways (R_0 _> CDE_0 _+ CIE_0_), low and high EGF-stimulations lead to two different steady-states, respectively. In steady-state I, the receptor internalizes primarily via CDE, whereas in steady-state II it is equally partitioned between CDE and CIE.

Initial values	Steady-state	CIE-internalization
EGF_0 _< CDE_0_	I	low
CDE_0 _+ CIE_0 _< EGF_0_	II	high

### Steepness of switch effect

An ultrasensitive response of a signaling system is characterized by a low, or damped response up to a certain threshold of stimulus, followed by an abrupt increase towards maximal response when this threshold is exceeded [23,26,27,50]. It has been derived to result from positive feedback or multisite-modification [24,25,50,52,53]. Ultrasensitivity has also been shown to arise in (de-)modification cycles if the enzymes operate near saturation [31], which makes the mechanism very sensitive to small parameter changes [26], if the abundance levels of unmodified substrate and enzyme are sufficiently high (ultrasensitization, [33]) or if the enzyme is inhibited [28].

**Figure 5:**
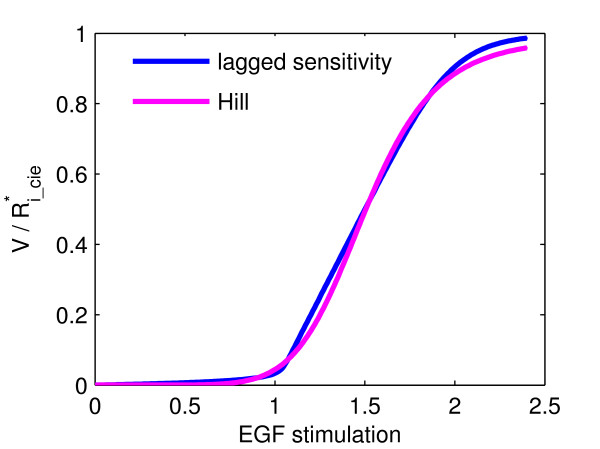
**Approximability of switch-effect by Hill-curve**. Comparison between Hill-type response and the described switch effect. Plotted are steady-state values of R_i_cie _or reaction velocity V (Hill-kinetics). Parameter and initial values used were the same as for Fig. 3C. Hill-parameters extracted from the stimulus-response curve were: h = 7.5, V_*max *_= 1.0, K_*m *_= 1.4.

To characterize the steepness of the here discussed mechanism, we compared its response to a Hill-type reaction (see Methods). Figure 5 shows the reaction velocity V of the Hill-formula, compared to ${\text{R}}_{\text{i\_cie}}^{*}$-production in our model (stimulus-response curve) as a function of EGF-stimulation. To generate the stimulus-response curve, we chose the same parameter set as for Fig. 3C as a reference. From this curve we extracted the Hill-coefficienth, V_*max *_and K_*m *_to compute the corresponding Hill-curve, which will be used as a reference curve later on. The Hill-coeffcient is a measure of how much the input has to be increased in order to raise the response from 10% to 90% of its maximal value [28]. Stimulus-response curves with Hill-coefficients of 5 or higher are generally considered ultrasensitive [25,26,28]. The Hill-coefficient obtained for the stimulus-response curve shown in Figure 5 is 7.5.

### Robustness of solution

It has been argued that biologically functional modules or pathways need to be robust against variations of reaction parameters and protein concentrations in order to ensure proper functioning [54,55]. The concept of robustness refers to the 'purpose' of a certain module or pathway: it is expected that intracellular network structures have undergone an evolution that guarantees their proper functioning independently of precise parameter values [56].

To transfer this concept to the question of receptor sorting into alternative pathways, we asked to what extent the functioning of the here described module depends on exact parameter or initial values. As functioning we defined the clear-cut sorting of the receptor into distinct routes, namely CDE at low, respectively CDE and CIE at high ligand concentrations.

**Figure 6:**
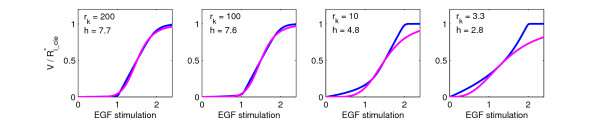
**Robustness of switch-effect for parameter variations**. Comparison between stimulus-response curve (with Hill-coefficient h) and corresponding Hill-curve for selected r_k _values. Here, k_cde _= 1.0 and k_cie _was varied. Plotted are steady-state values of R_i_cie _(blue) or reaction velocity V (Hill-kinetics, magenta).

**Figure 7:**
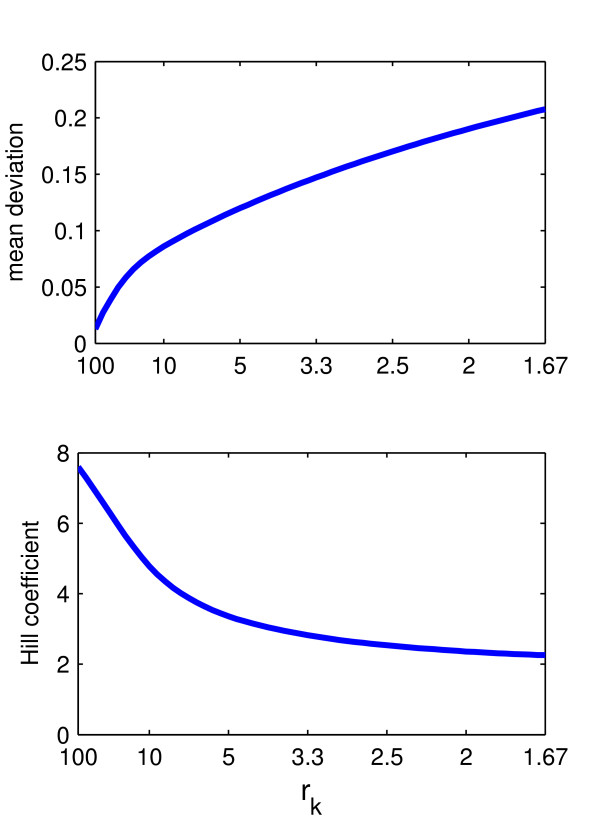
**Deviation from Hill-curve**. Mean deviation from reference Hill-curve and Hill-coefficient of stimulus response curves for varying ${\text{r}}_{\text{k}}=\frac{{\text{k}}_{\text{cde}}}{{\text{k}}_{\text{cie}}}$. Here, k_cde _= 1.0 and k_cie _was varied between 0.01 (r_k _= 100) and 0.6 (r_k _= 1.67). All other parameter and initial values were as in Fig. 3C.

The key parameter and initial concentrations that affect the strength of the switch effect are the initial concentrations of CDE- and CIE-adaptors as well as ${\text{r}}_{\text{k}}=\frac{{\text{k}}_{\text{cde}}}{{\text{k}}_{\text{cie}}}$. Obviously, the ultrasensitive response will be steeper the higher the difference in binding kinetics for the respective pathways is, i.e. the greater r_k_. We systematically varied r_k _and from each thus obtained stimulus response curve of ${\text{R}}_{\text{i\_cie}}^{*}$-values extracted the Hill-coefficient h as a measure of steepness (see Methods). The Hill-coefficients varied from 2.8 (r_k _= 3.3) to 7.7 (r_k _= 200) as shown in Figure 6. We also computed the mean deviation between the obtained stimulus response curves and the reference Hill-curve (Figure 7), showing that for considerable variations of r_k _the stimulus-response curve remains approximable by a Hill-curve.

**Figure 8:**
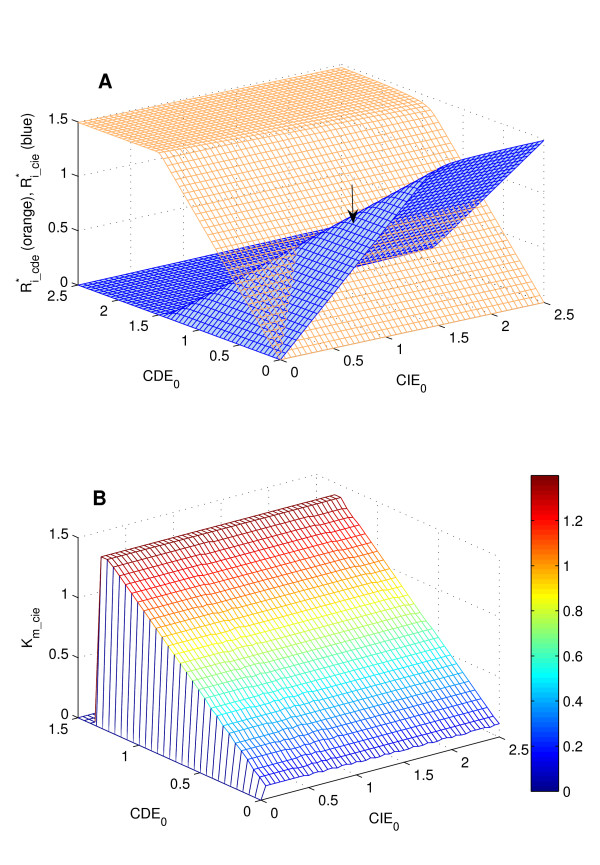
**Dependence of switch effect on abundance levels of endocytosis adaptors**. Plotted are ${\text{R}}_{\text{i\_cie}}^{*}$ (blue) and ${\text{R}}_{\text{i\_cde}}^{*}$ (orange) as a function of CDE_0 _and CIE_0_. EGF_0 _= R_0 _= 1.5. See text for interpretation.

In Figure 8A we plotted the steady-state values of R_i_cde _and R_i_cie _(V_*max *_values of the stimulus-response curves) as a function of initial adaptor values CDE_0 _and CIE_0_. Here, R_0 _and EGF_0 _were chosen 1.5.

Consider the curve for ${\text{R}}_{\text{i\_cie}}^{*}$ (blue). For initial adaptor values such that CDE_0 _+ CIE_0 _< R_0_, EGF_0 _(see arrow), the curve is largely independent of CDE_0 _and increases linearly as a function of CIE_0 _up to the threshold of 1.5. The independence of CDE_0 _reflects the fact that if neither receptors nor ligand are limiting for the internalization reaction (steady-state class II), the steady-state amount of receptor internalized via CIE is solely dependend on the initially available number of CIE-adaptors. Outside of this range, ${\text{R}}_{\text{i\_cie}}^{*}$ decreases with increasing CDE_0 _and becomes zero for CDE_0 _> 1.5(cf. Figure 3A). ${\text{R}}_{\text{i\_cie}}^{*}$ (orange curve) is largely independent of CIE_0 _and increases linearly with CDE_0 _up to the threshold of 1.5 when ligand or receptor number become limiting.

The threshold of CIE-internalization (K_*m *_of the stimulus-response curves) is independent of CIE_0 _(for CIE_0 _> 0) and is equal to CDE_0 _as shown in Figure 8B.

### Role of Receptor modifications

It is well-known that ligand-induced receptor modifications in the form of phosphorylation and/or ubiquitination play a functional role in signaling and contribute to the specificity of adaptor-binding. However, our analysis focused on the question, whether for a precise sorting of receptors into the two alternative endocytosis pathways discussed here a *discriminative *modification is necessary. In this light, R_EGF, which in our model indicates the activated receptor species capable of interacting with the endocytosis adaptors, could also represent an already modified form of the receptor. To illustrate this point, we extended the model as given in equations (1.1 – 1.7) to include the binding of the ubiquitin-ligase Cbl followed by ubiquitination of the receptor. The equations read

(2.8) d(EGF)/dt = -k_f _* EGF * R + k_r _* R_EGF

(2.9) d(R)/dt = -k_f _* EGF * R + k_r _* R_EGF

(2.10) $$\begin{array}{lll} \text{d}(\text{R\_EGF})/\text{dt} & = & {\text{k}}_{\text{f}}*\text{EGF}*\text{R}-{\text{k}}_{\text{r}}*\text{R\_EGF}\\ & & -{\text{k}}_{\text{onCbl}}*\text{R\_EGF}*\text{Cbl} \end{array}$$

(2.11) + k_offCbl _* R_EGF_Cbl

(2.12) $$\begin{array}{lll} \text{d}(\text{Cbl)}/\text{dt} & = & -{\text{k}}_{\text{onCbl}}*\text{R\_EGF}*\text{Cbl}\\ & & +{\text{k}}_{\text{offCbl}}*\text{R\_EGF\_Cbl}\\ & & +{\text{k}}_{\text{catCbl}}*\text{R\_EGF\_Cbl} \end{array}$$

(2.13) $$\begin{array}{lll} \text{d}(\text{R\_EGF\_Cbl)}/\text{dt} & = & {\text{k}}_{\text{onCbl}}*\text{R\_EGF}*\text{Cbl}\\ & & -{\text{k}}_{\text{offCbl}}*\text{R\_EGF\_Cbl}\\ & & -{\text{k}}_{\text{catCbl}}*\text{R\_EGF\_Cbl} \end{array}$$

(2.14) $$\begin{array}{lll} \text{d}(\text{R\_EGF\_Ub)}/\text{dt} & = & {\text{k}}_{\text{catCbl}}*\text{R\_EGF\_Cbl}\\ & & -{\text{k}}_{\text{cde}}*\text{R\_EGF\_Ub}*\text{CDE}\\ & & -{\text{k}}_{\text{cie}}*\text{R\_EGF\_Ub}*\text{CIE} \end{array}$$

(2.15) d(CDE)/dt = - k_cde _* R_EGF_Ub * CDE

(2.16) d(CIE)/dt = - k_cde _* R_EGF_Ub * CIE

(2.17) d(R_i_cde_)/dt = k_cde _* R_EGF_Ub * CDE

(2.18) d(R_i_cie_)/dt = k_cie _* R_EGF_Ub * CIE

Here, k_onCbl _and k_offCbl _are the rate constants for the association and dissociation of Cbl to ligand-bound receptor R_EGF, and k_catCbl _is the rate of the ubiquitination step.

**Figure 9:**
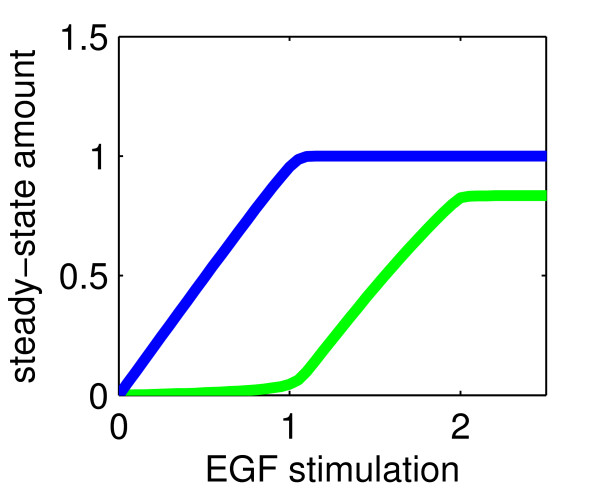
**Inclusion of Receptor Modifications**. Plotted are steady-state values of R_i_cde _(blue) and R_i_cie _(green) as a function of EGF-stimulation (EGF_0_) for the extended model, including receptor ubiquitination. The switch effect is preserved under the assumption of receptor modifications.

Again, we tested whether the assumption that both pathways consume the thus, i.e. equally, modified form of receptor would comply with the observation of an ultrasensitive response of CIE-internalization to increasing EGF-stimulation. In Figure 9 we plotted the steady-state values of receptor internalized via CDE(R_i_cde_, blue) and CIE (R_i_cie_, green), respectively. Clearly, the response is comparable to the results of the simpler model (Figure 3C), proving that the existence of receptor modifications prior to internalization does not affect our results. The rate constants and initial values : k_f _= 1.0, k_r _= 0.01, k_onCbl _= 1.0, k_offCbl _= 0.01, k_catCbl _= 1.0, k_cde _= 1.0, k_cie _= 0.01, R_0 _= 2.0, Cbl_0 _= 2.0, CDE_0 _= 1.0, CIE_0 _= 1.6. All other initial values are zero.

### Model extension for more than two Pathways

For EGF receptor, evidence for the existence of more than just two independent endocytosis pathways has been given [19,57]. To our knowledge, EGF-concentration dependence has not been shown. Here, we describe an extension of the above model to more than two pathways which might play a role in other contexts.

**Figure 10:**
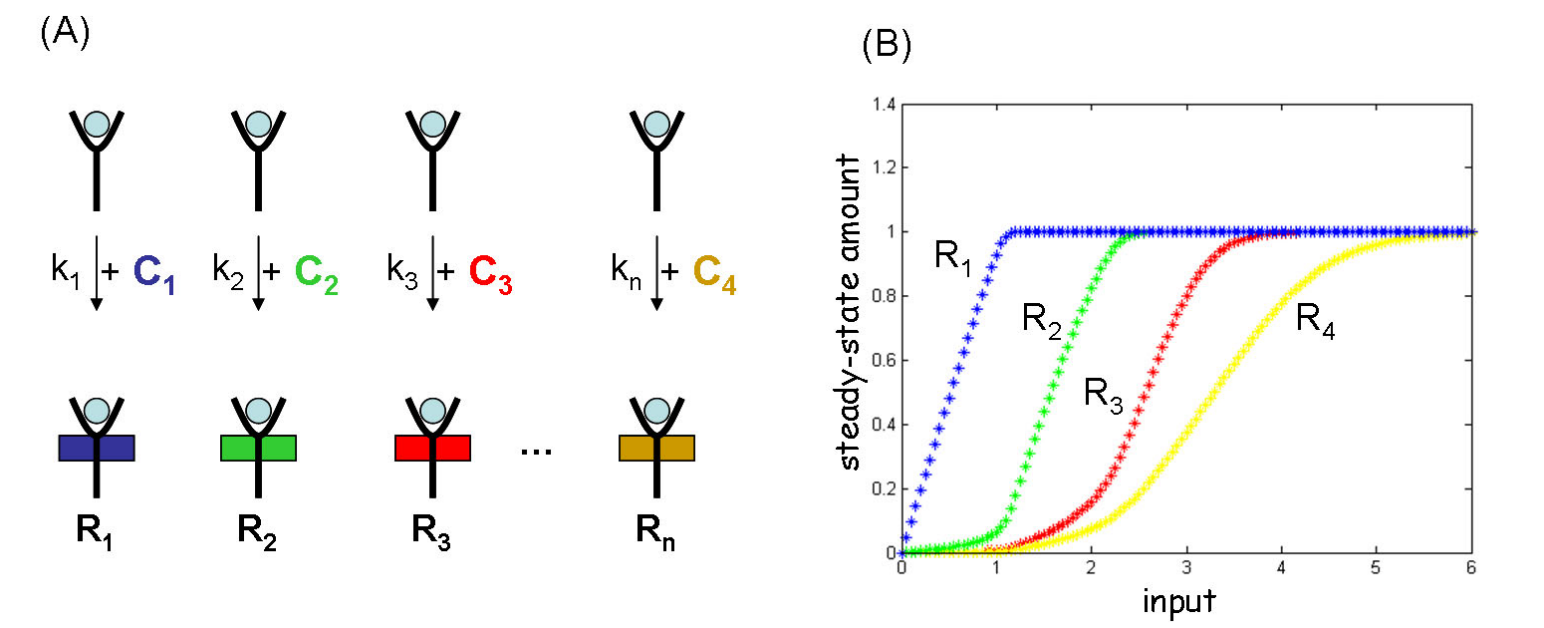
**Model extension to n > 2 pathways**. (A) Extension of the model for *n *> 2 cases. (B) Simulation for *n *= 4 cases. Plotted are steady-state amounts of R_*i*_, *i *= 1, 2, 3, 4. Rate constants and initial values used: k_f _= 1.0, k_r _= 0.01, k_1 _= 2.0, k_2 _= 0.05, k_3 _= 0.005, k_4 _= 0.001, R_0 _= 9.0, C_1 _= 1, C_2 _= 1, C_3 _= 1, C_4 _= 1. All other initial values were zero.

Suppose that *n *> 2 pathways branch off from one activated signaling molecule R_*L *_(playing the role of ligand-bound receptor R_EGF). Assume that R_*L *_can bind to n different types of adaptor molecules C_*i *_with reaction rates k_*i*_, i = 1,2, ..., *n *where k_*i *_>> k_*i *+ 1_, i = 1,2, ... *n *- 1. Then the sorting effect based on ultrasensitivity is extended to m cases if $R_{L_{0}}\geq \sum_{i=1}^{m}C_{i_{0}}$, m ≥ *n*, with X_0 _denoting the initial value of molecule class X. The effect is illustrated in Fig. 10B for *n *= 4. The differential equations read:

d(L)/dt = -k_f _* L * R + k_r _* R_L_

d(R)/dt = -k_f _* EGF * R + k_r _* R_L_

$$\begin{array}{lll} \text{d}({\text{R}}_{\text{L}})/\text{dt} & = & {\text{k}}_{\text{f}}*\text{L}*\text{R}-{\text{k}}_{\text{r}}*{\text{R}}_{\text{L}}-{\text{k}}_{1}*{\text{R}}_{\text{L}}*{\text{C}}_{1}-{\text{k}}_{1}*{\text{R}}_{\text{L}}*{\text{C}}_{2}\\ & & -{\text{k}}_{\text{3}}*{\text{R}}_{\text{L}}*{\text{C}}_{\text{3}}-{\text{k}}_{\text{4}}*{\text{R}}_{\text{L}}*{\text{C}}_{\text{4}} \end{array}$$

d(C_i_)/dt = -k_i _* R_L _* C_i_

d(R_i_)/dt = -k_i _* R_L _* C_i_

*i *= 1, 2, 3, 4

## Discussion

Our analysis addresses the experimentally observed dependence of endocytosis on EGF-concentration [5,21]. We propose an ultrasensitive sorting mechanism for EGF receptor internalization which does not require a discriminative receptor modification and give a systematic description of the parameter requirements to achieve proper sorting. We derive analytically the existence of alternative steady-states as well as conditions on the abundance levels of receptors, ligand and endocytic adaptors to reach these states.

Referring to previous models of sigmoidal responses based on cooperativity, for the EGF receptor in particular a cooperative binding effect of the ubiquitin-ligase Cbl during the ubiquitination reaction has been proposed to be necessary for the observed switch-effect of CIE internalization [44]. Our analysis explains how imposing weaker assumptions on the internalization machinery, namely that the two pathways are entered with distinct affinities, is sufficient to explain the observed switch-effect. Importantly, it is pointed out how by varying the abundance levels of active receptors or endocytic adaptors, cells may modulate their response to incoming EGF-stimulations: depending on the initially available receptors or adaptors, the distribution of internalized receptors can be different for one and the same EGF concentration (cf. Figure 3, Figure 8A).

The lack of knowledge about the true parameter/initial values was accounted for by systematic variations over broad numerical ranges. The robustness of the switch-effect to exact parameter values argues for the plausibility of the introduced mechanism.

Mathematical models addressing the problem of receptor sorting into alternative endocytosis pathways do not currently exist. Previously proposed hypotheses based on experimental data only have not been able to give a satisfying answer to this question [9,12,18-21,58]. Generally, the problem is considered at the 'single-molecule-level' : a single receptor is envisioned, which is thought to enter either the CDE- or the CIE-route (see Figure 1). This picture misleadingly implies the necessity of a discriminative receptor modification.

Instead of thinking in terms of individual entities, we propose to consider the dynamical properties of a system of interacting molecule populations. Applying methods from the theory of dynamical systems, we were able to conceive that an increase in the ligand concentration above the capacity of the CDE-pathway qualitatively alters the system behaviour by enforcing an alternative steady-state (Fig. 3, Table 1). Our model states that an abrupt, switch-like start of CIE occurs if the extracellular EGF concentration exceeds the capacity of the CDE machinery. This proposes an interesting implication of the regulation of receptor sorting: the cell achieves the switch-effect 'for free' since no extra cost has to be invested into a discriminative receptor modification. It can be assumed that cells have evolved to optimize energy efficiency [59]. Utilizing the kind of dynamics introduced here, where just one form of receptor is consumed by both pathways, could thus constitute an evolutionary advantage.

A second major observation we draw from the model is that the described mechanism provides a means for an individual cell to sense its surrounding medium: clathrin-independent endocytosis is switched on precisely when the extracellular ligand concentration exceeds the number of CDE-adaptors. One might interpret this mechanism as a protein module, i.e. a small interaction network acting as a computational element, whose purpose is to store and process information [51,60,61].

One might argue that the simplicity of the model impairs its abilitiy to uncover unanticipated results. While an initiation of clathrin-independent endocytosis upon saturation of the clathrin pathway might have been proposed without mathematical modeling, the unexpected steepness of this switching-behaviour as well as its robustness could not have been revealed by intuition alone [9,18,19]. Furthermore, we showed that extending the model by allowing a modification of the receptor does not increase the steepness of the response. Thus, we conclude that a modification of receptor is not required to discriminate between the pathways. This does notably not exclude the possibility that a modification of the receptor might have been chosen by nature to ensure proper endocytic sorting.

Finally, we want to emphasize that the generality of our model makes it applicable to ultrasensitivity in signaling processes other than the here discussed problem of receptor sorting. This is highlighted in the potential of the model to be extended to more than two overlapping signaling pathways (cf. Figure 9)

## Conclusion

We describe the dynamical consequences of an interaction motif, whose molecular basis has already been well established experimentally and discuss its applicability to endocytic sorting of the EGF receptor. We give a simple, yet mathematically sound explanation how cell variation of endocytic sorting results from modulating abundance levels of involved cellular molecules. In the light of the difficulty to experimentally identify a discriminative receptor modification, our analysis points to the possibility of a systems-level regulation of endocytic sorting. The natural extensibility of the model to more than two cases may prove applicable in other signaling contexts.

## Methods

### Numerical Simulations and Steady-State Analysis

To numerically solve the systems of equations (1.1 – 1.7) we used the MATLAB ODE15s function. The existence of distinct classes of steady-states was derived as follows: The system of ODEs exhibits four independent equations, namely equations (1.1), (1.3), (1.4) and (1.5). To obtain the values of the variables that represent a steady-state, we simultaneously set these equations equal to zero and solved for the variables.

Setting equations (1.4) and (1.5) equal to zero yields

(1.4a) -k_cde _* R_EGF *CDE = 0

(1.5a) -k_cie _* R_EGF *CDE = 0

Since all kinetic constants are assumed to be positive, one derives that either R_EGF* or both CDE* and CIE* have to be zero. Consider the case that R_EGF* = 0. Then, from setting equations (1.1) and (1.3) equal to zero, i.e.

(1.1a) k_f _* EGF * R - k_r _* R_EGF = 0

(1.2a) $$\begin{array}{l} {\text{k}}_{\text{f}}*\text{EGF}*\text{R}-{\text{k}}_{\text{r}}*\text{R\_EGF}\\ -{\text{k}}_{\text{cde}}*\text{R\_EGF}*\text{CDE}\\ -{\text{k}}_{\text{cie}}*\text{R\_EGF}*\text{CIE}=0 \end{array}$$

it follows that either R* or EGF* have to be zero. We have thus derived steady-state class I (see Results). Consider now the second case derived from equations (1.4a) and (1.5a), namely that CDE* = 0 and CIE* = 0. Substituting these values into equation (1.3), one obtains

(1.3a) $${\text{R\_EGF}}^{*}=\frac{{\text{k}}_{\text{f}}}{{\text{k}}_{\text{r}}}{\text{EGF}}^{*}*{\text{R}}^{*},$$

which also leaves equation (1.1) equal to zero. This second set of values represents steady-state class II (see Results).

### Approximability by Hill-curve

In order to assess the steepness of the derived switch-effect, we compared the obtained stimulus-response curve to the switch-effect described by the Hill-formula. This formula was originally introduced as a phenomenological model to describe the binding of oxygen to hemoglobin [62] and later often used to for cooperative enzyme reactions:

(3.1) $$\text{V}=\frac{{\text{V}}_{\text{max}}*{\text{x}}^{\text{h}}}{{\text{K}}_{\text{m}}^{\text{h}}+{\text{x}}^{\text{h}}}.$$

Here, V denotes reaction velocity, V_max _the maximal velocity, x the substrate concentration, K_m _the substrate concentration where half-maximal velocity is reached and h, the Hill-coefficient, represents the steepness of the response.

In order to investigate the sensitivity of the switch-effect of CIE-internalization on specific parameter values, we adapted a procedure applied in [26]. We systematically varied reaction parameters and initial values of the model (equations 1.1 – 1.7) using equidistant sampling points. For each thus obtained set we computed the stimulus-response curve and extracted the Hill-coefficient as $\text{h}=\frac{\log81}{\log({\text{x}}_{0.9})\log({\text{x}}_{0.1})}$[25]. Here, x_0.1 _and x_0.9 _are the EGF-concentrations where 10% or 90% of the maximal responseis reached, espectively.

To evaluate the approximability of the stimulus-response curve by the Hill-equation, we computed the mean deviation between the obtained stimulus-response curves and a reference Hill-curve. In this way we formally determined a broad range of parameter values for which an ultrasensitive response of CIE-internalization occurs (Fig. 5 and Fig. 6).

## List of Abbreviations

The following abbreviations are used: CDE – Clathrin dependent endocytosis, CIE – Clathrin independent endocytosis, EGF – Epidermal Growth Factor, EGFR – Epidermal Growth Factor Receptor, mono-Ub – mono-ubiquitination

## Authors' contributions

HSG designed the study, performed the simulations and mathematical analysis and wrote the manuscript. IV designed the study, helped with the mathematical analysis and wrote the manuscript. DH proposed the problem of endocytic sorting for mathematical modeling and helped design the study. ID proposed the problem of endocytic sorting for mathematical modeling and helped design the study. RE proposed the problem of endocytic sorting for mathematical modeling, designed the study and wrote the manuscript.
